# Development of a Hybrid Intelligent Process Model for Micro-Electro Discharge Machining Using the TTM-MDS and Gaussian Process Regression

**DOI:** 10.3390/mi13060845

**Published:** 2022-05-28

**Authors:** Yanyan Chen, Xudong Guo, Guojun Zhang, Yang Cao, Dili Shen, Xiaoke Li, Shengfei Zhang, Wuyi Ming

**Affiliations:** 1 College of Mechanical Engineering, Yellow River Conservancy Technical Institute, Kaifeng 475004, China; chyy1106@126.com; 2 Mechanical and Electrical Engineering Institute, Zhengzhou University of Light Industry, Zhengzhou 450002, China; gggxudong@163.com (X.G.); lixiaoke@zzuli.edu.cn (X.L.); 3 Guangdong Provincial Key Laboratory of Digital Manufacturing Equipment, Guangdong HUST Industrial Technology Research Institute, Dongguan 523808, China; guojun_zhang_stu@163.com; 4 School of Mechanical Electronics and Automobile Engineering, Zhengzhou University of Technology, Zhengzhou 450002, China; shendili@163.com (D.S.); zhangshengfei2021@163.com (S.Z.)

**Keywords:** micro-EDM, process modeling and simulation, molecular dynamics simulations (MDS), Gaussian process regression (GPR)

## Abstract

This paper proposed a hybrid intelligent process model, based on a hybrid model combining the two-temperature model (TTM) and molecular dynamics simulation (MDS) (TTM-MDS). Combined atomistic-continuum modeling of short-pulse laser melting and disintegration of metal films [Physical Review B, 68, (064114):1–22.], and Gaussian process regression (GPR), for micro-electrical discharge machining (micro-EDM) were also used. A model of single-spark micro-EDM process has been constructed based on TTM-MDS model to predict the removed depth (RD) and material removal rate (MRR). Then, a GPR model was proposed to establish the relationship between input process parameters (energy area density and pulse-on duration) and the process responses (RD and MRR) for micro-EDM machining. The GPR model was trained, tested, and tuned using the data generated from the numerical simulations. Through the GPR model, it was found that micro-EDM process responses can be accurately predicted for the chosen process conditions. Therefore, the hybrid intelligent model proposed in this paper can be used for a micro-EDM process to predict the performance.

## 1. Introduction

Electrical discharge machining (EDM) is a non-contact, non-traditional manufacturing process. In this process, electrically conductive materials can be removed by the electrical spark. In the current manufacturing scenario, it makes EDM an important processing tool in the manufacture of molds, complex-shaped dies, and critical parts used in shipbuilding, mold manufacturing, automobiles, aerospace, and other industrial applications [1,2]. In recent years, the working limits of EDM have been extended to micron scale, namely micro-EDM, based on newly developed relevant advanced technologies and devices, such as manufacturing of micro-electrodes and high-performance power supplies, etc. [3].

In the EDM process, the discharge phenomenon occurs in a narrow space that is filled with dielectric fluid in a few microseconds. Evaporation and melting of the material result in the removal of material from both electrodes owing to the occurrence of consecutive sparks [4]. In practice, because the EDM process with plasma production is considerably complex, it is hard to reveal the mechanism of material removal by the experiment [5]. Thus, the development of an EDM process model for the prediction of surface quality and MRR has become a hotspot in the study of EDM.

Nowadays, the influences of process parameters such as electrical and non-electrical parameters on EDM performance are being investigated [6,7,8,9,10,11,12]. In these studies, it is shown that the electrical parameters play a dominant role in the performance. Electrode wear rate (EWR) has been discussed by some scholars [13,14,15,16], and the results show that electrical parameters are the main factors that impact the performance of EDM. From the literature [17], it can be drawn that cutting performance indicators such as MRR and surface roughness (Ra) are strongly related to the characteristics of EDM discharge pulses. Noticeably, these studies on the EDM process are mainly based on experimental analysis.

However, the abovementioned studies are merely applicable to specific machines, tool-work materials, and workshop conditions. Considering that the key problem of EDM and micro-EDM is the accurate remanufacturing of complex tool shapes and the economy of machining, the main aim of this paper is to develop a hybrid intelligent process model for micro-EDM to accurately predict the RD and MRR by integrating the combined atomistic-continuum model, which is a hybrid model of a two-temperature model and molecular dynamics simulation (TTM-MDS) [18], and the Gaussian process regression (GPR). Then, the finishing capability and process productivity can be improved by using the selected optimum process parameters from this model. The rest of this paper is organized as follows. Section 2 presents an overview of the developed integrated process model for micro-EDM using TTM-MDS and GPR. Section 3 describes the details of the construction of the TTM-MDS model. Section 4 discusses the influence of various input process parameters on process performance and Section 5 presents the development of a GPR-based process model. Section 6 mainly discusses the experimental results from the view of energy input intensity and crater shape, and Section 7 summarizes the conclusions and outlooks from this work.

## 2. Integrated Process Model for Micro-EDM

It is very difficult to elucidate the mechanism of material removal and crater formation through experiments because of the complex physical phenomenon in the EDM process. It is a feasible method to elucidate the mechanism of material removal from the perspective of a hot atom movement. The development of an EDM process model for the prediction of surface quality and MRR has become a hotspot in the study of EDM. Molecular dynamics can reveal the rules of an EDM process from microscopic and dynamic simulations. Molecular dynamics is the most common method used to study a classical multi-particle system, which is a deterministic simulation method. However, the molecular dynamics model is complicated, and the simulation requires high performance on computer equipment, and long running time. Therefore, the GPR proxy model is based on molecular dynamics. The response of a GPR model is fast and the time of a single simulation only takes a few seconds. A GPR model can predict the material removal rate and crater depth effectively.

**Figure 1:**
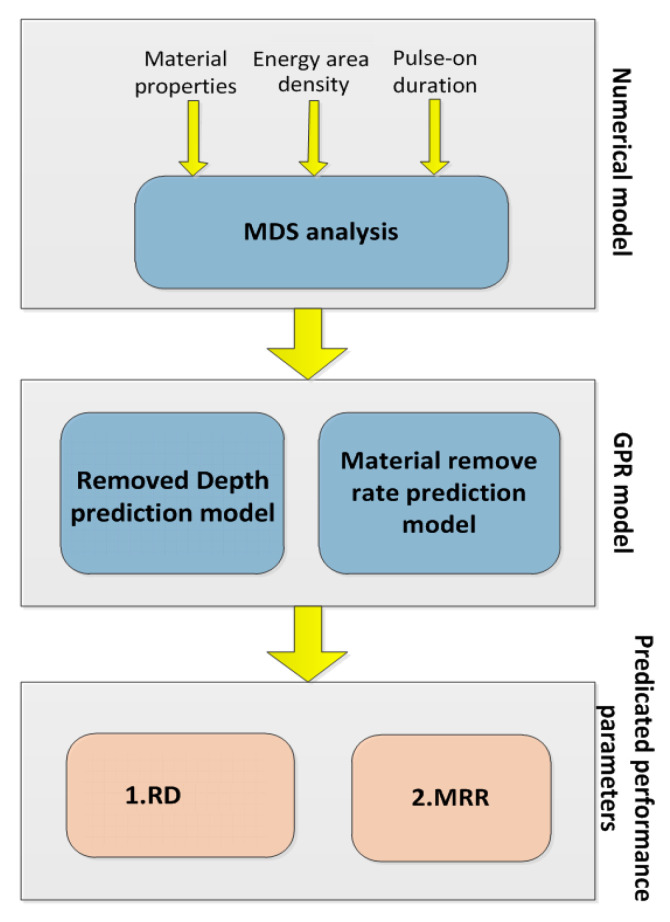
A hybrid integrated process model for Micro-EDM.

Figure 1 depicts the overall methodology used to create this micro-EDM integrated process model. There are two main stages in the proposed method. Molecular dynamics analysis of material properties, energy density, and pulse duration were used to establish the mathematical model in the first stage. In the second stage, the GPR model was proposed for micro-EDM to establish a relationship between input process parameters (energy area density and pulse-on duration) and process responses (RD and MRR). Using the data generated from the numerical simulations, the GPR model was trained, tested, and tuned. Finally, the GPR model can accurately predict the micro-EDM process responses under the chosen process conditions.

## 3. Numerical Modeling of the Micro-EDM Process

### 3.1. Micro-EDM Model

The dynamics behaviors and micro-structure evolution of the material can be described by the MDS to simulate the micro-EDM, which are essential to reveal the machining mechanism [19]. Considering that when a huge amount of thermal energy from the discharge channel is input intensely, the great thermal in-equilibrium between the lattice system and the electron system of the electrode material will arise. The process will take hundreds of femtoseconds to tens of picoseconds for the lattice system and the electron system to reach thermal equilibrium. The characteristic time scale of MDS is femtosecond and the simulation time length is usually less than 1 ns, so the special dealings have to be conducted to cope with the thermal in-equilibrium between the two systems.

**Figure 2:**
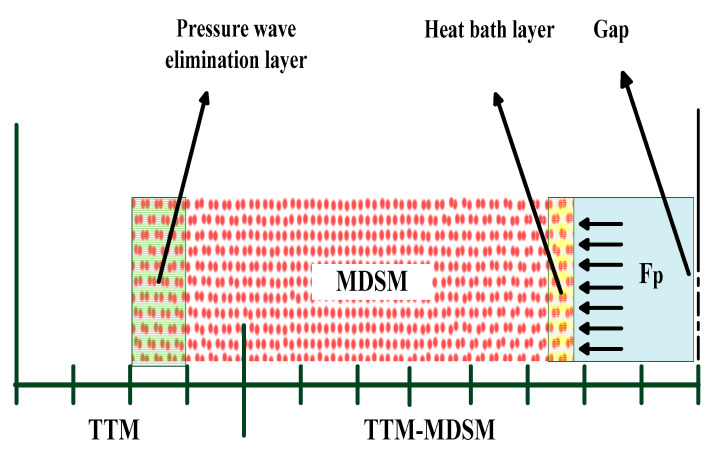
Schematic diagram of the combined atomistic-continuum model.

The modeling method proposed by Ivanov [18] is used to solve the issue mentioned above, and the schematic diagram of this combined atomistic-continuum model is shown in Figure 2. In this method, the two temperature models (TTM) and the MDS model (MDSM) are integrated. The TTM is used to deal with the thermal equilibrium between the electron system and the lattice system, and the MDSM is used to describe the dynamical behavior of material. In the TTM, two heat transfer subsystems for the electron system and the lattice system are respectively constructed, and the governing equations are as follows,

(1) $$\;C_{e}\left(T_{e}\right)\frac{\partial T_{e}}{\partial t}=\nabla \left[K_{e}\left(T_{e},T_{l}\right)\nabla T_{e}\right]-G\left(T_{e}-T_{l}\right)+H_{e}\left(r,t\right)$$

(2) $$\;\;C_{l}\left(T_{l}\right)\frac{\partial T_{l}}{\partial t}=\nabla \left[K_{l}\left(T_{l},T_{l}\right)\nabla T_{l}\right]-G\left(T_{e}-T_{l}\right)+H_{l}\left(r,t\right)$$

where *C* is the heat capacity, *K* the is thermal conductivity, *G* is the electron-lattice coupling factor, *H* is the heat resource, *r* is the position vector, while the subscripts *e* and *l* denote the corresponding material parameters and variables of electron system and lattice system, respectively. In our model, bulk Cu is taken as the target, thus, where *K*_e_ = *K*_l_ = 97 J.m^−3^.K^−2^, *G* = 1 × 10^17^ J.m^−3^.s^−1^.K^−1^, *C*_l_ = 3.5 × 10^6^ J.m^−3^.K^−1^ [20]. Thermal energy is not input through the TTM, therefore, *H*_e_ = *H*_l_ = 0. The initial and boundary conditions are,

$$\begin{array}{l} \mathrm{IC}:\;t=0,\forall \left(r\right),T=293\;K\;\\ \mathrm{BC}:\;T=273\;K,\;\mathrm{for}\;r\in \partial \Omega \; \end{array}$$

where *∂*Ω denotes the boundary of the whole region of TTM. To minimize the influence of the boundary condition, the length of the TTM is set to be 4 µm, much longer than the possible referred length scale in simulation. In addition, finite difference method is employed to solve Equations (1) and (2).

As shown in Figure 2, the MDSM is embedded into the TTM. Assuming that the interaction between two electrodes is not considered. The MDSM is composed of 259,200 copper atoms and with dimensions of 3.25 × 3.25 × 289.20 nm. The embedded atom method potential proposed by Wadley [21] and Zhou [22] is used to describe the interaction between atoms. Free boundary condition is applied to the surface contacting to the gap and the bottom surface, and periodic boundary condition is applied to the other four lateral surfaces. In addition, the atoms in the bottom region have been specially treated to eliminate the reflection of pressure wave from the upper surface, which is produced by the abruptly input thermal energy [23].

Coupling between the TTM and the MDSM is dealt with by adding an extra force to the motion equation of each atom in the MDSM to take the exchange of thermal energy between the two models into account,

(3) $$m_{j}\frac{d^{2}r_{j}}{dt^{2}}=F_{j}+\xi m_{j}v_{j}^{T}$$

where *m_j_* is the matter of the *j*_th_ atom, *F*_j_ is the resultant force from the regular MDS calculation, *v*_j_*^T^* is the thermal velocity, and *ξ* is the influence coefficient which determines the strength of the heating effect which the electron system imposes on the lattice system. The one in the *N*_th_ TTM difference cell is expressed by,

(4) $$\xi_{N}=\frac{1}{n}\sum_{k=1}^{n}GV_{N}(T_{e}^{k}-T_{l})/\sum_{j}m_{j}{\left(v_{j}^{T}\right)}^{2}$$

where *n* is the times of the MDS time step to the TTM time step, and *V_N_* is the volume of the *N*_th_ TTM difference cell [18].

The influence of the discharge channel on the electrode includes two parts, one is the thermal effect due to the high temperature, and the other is the mechanical effect due to the high pressure. To take the thermal effect into account, a heat bath proposed by Berendsen [24] with temperature ranging from 5070 K to 18,107 K is applied to the 5 nm in thickness area label by “Heat bath layer” (HBL) as shown in Figure 2. To count the mechanical effect, an extra force *F*_p_ pointing straight to the electrode is imposed on each atom in the HBL. The *F*_p_ is calculated by,

(5) $$F_{p}=pA/n$$

where *p* is the pressure, *A* is the cross-section area of the HBL, and *n* is the number of atoms in the HBL. The quantity of *p* is dependent on the applied temperature, and determined according to Zhao et al. [25] on the assumption that no expansion occurs to the dielectric water.

### 3.2. Model Results

**Figure 3:**
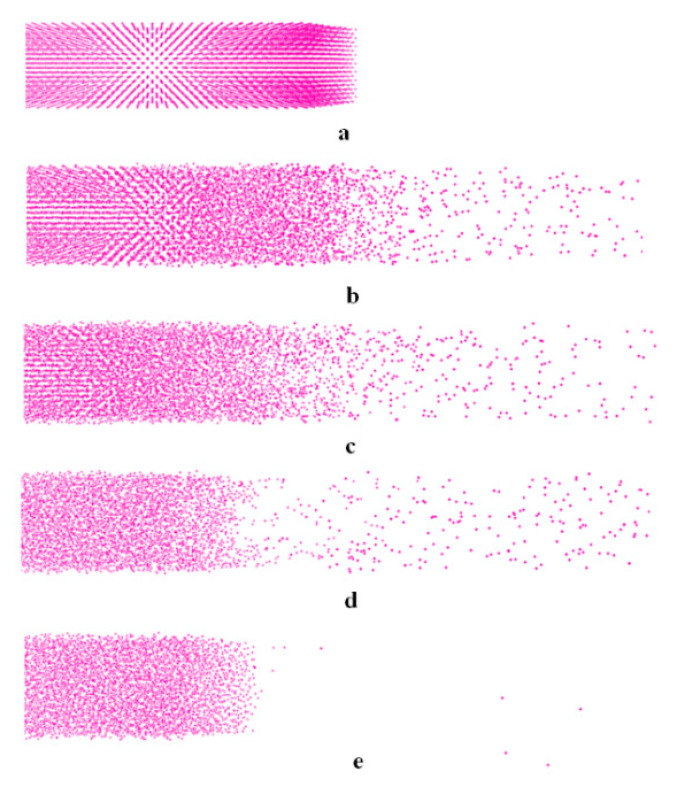
Snapshots in simulation of a bulk Cu machined by single electrical discharge in Micro-EDM process (Pe = 6.98 Kev.nm^−2^, T_on_ = 100 ps): (**a**) t = 0 ps; (**b**) t = 36 ps; (**c**) t = 60 ps; (**d**) t = 108 ps; (**e**) t = 168 ps.

The total input heat energy and material removal volume are the periodic boundary conditions on the four sides. MRR is converted into the average heat energy on the cross-section. The input of thermal energy per unit area is defined as energy area density (EAD). The thermal energy input per unit area in unit time is defined as energy flux (Q). Table 1 shows the experimental results from the simulation model of the micro-EDM process. In these experiments, EAD ranges from 1.04 to 9.04 KeV.nm^−2^; pulse-on duration (T_on_) has three levels: 30 ps, 100 ps, and 150 ps. Figure 3 shows the removing process of atoms with applied temperature of 13,761 K and T_on_ of 100 ps. After the emitting of discharge, atoms on the top surface melt and vaporize quickly, and about 36 ps later, the removing process starts. Thereafter, as thermal energy is continuously input, more and more atoms are removed, and about 168 ps after the end of the discharge, the removing process ceases.

It should be noted that atoms are removed mainly in the form of single atom or a small group of atoms instead of large clusters, which means that the main reason for removal of atoms is not mechanical spallation, but explosive disintegration of the overheated surface layers.

Different combination of EAD and T_on_ and the corresponding RD and MRR are shown in Table 1. For the case mentioned above (shown as the case of No. 9 in Table 1 below), at the end of a single electrical discharge, the RD is 4.04 nm, and the MRR is 0.43 nm^3^.ps^−1^. In the next section, the parameters on the micro-EDM process will be investigated specifically.

## 4. Parametric Studies on the Micro-EDM Process

**Figure 4:**
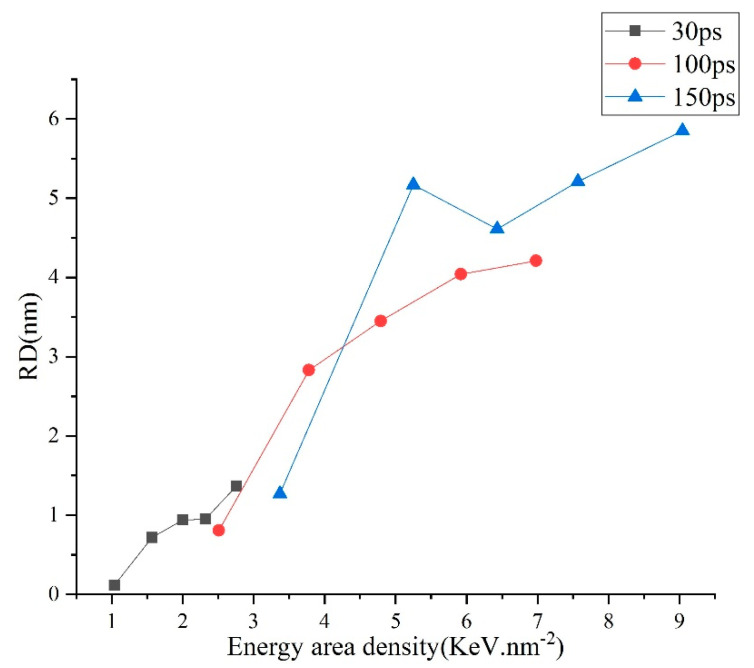
Profiles of the dependences of the removed depth on the energy area density.

**Figure 5:**
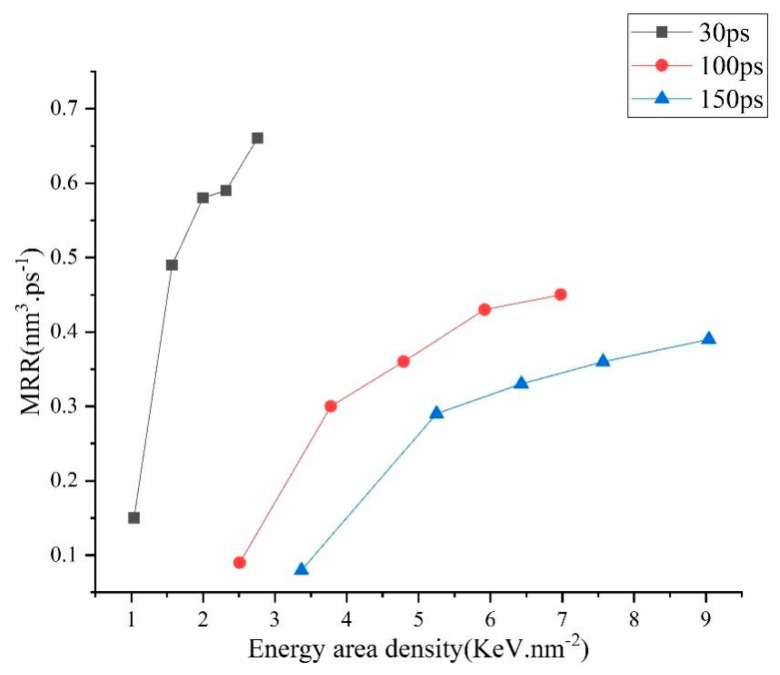
Profiles of the dependencies of the MRR on the energy area density.

**Figure 6:**
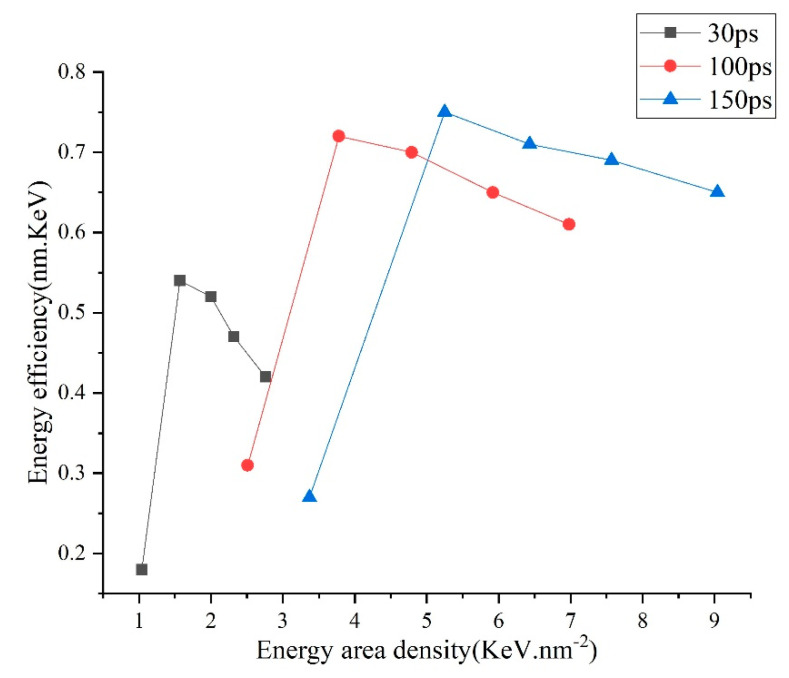
Profiles of the dependencies of the energy efficiency on the energy area density.

The results are shown in Figure 4, Figure 5 and Figure 6. Figure 4 describes the dependence of the RD on EAD. It can be seen that the removing process starts when the EAD increases to about 1 KeV.nm^−2^, and then the RD increases with the increase of the EAD overall. These results can be explained as follows. The possibility for an atom to escape the matrix is highly dependent on its kinetic energy (E_k_) which is used to overcome the impedance from atoms adjacent to the interested one and the pressure by the discharge channel. In certain circumstances, we can assume that when the kinetic energy of an atom exceeds a critical quantity, termed as E_C_, this atom will escape the matrix. At the start of the discharge, a certain amount of thermal energy (EAD_melt_) is needed to heat material and cause it to melt; in our model, EAD_melt_ is equal to 1 KeV.nm^−2^. When the EAD increases, material is heated to a higher temperature, and some atoms in the melted area manage to gain E_k_ higher than E_C_ and escape the matrix. The number of atoms (N_r_) obtained by E_k_ is more than that of E_C_. With the increase of EAD, the number of atoms (N_r_) removed is more. This phenomenon can be confirmed by the EDM or WEDM experiment. In Zhang’s study [10] regarding machining of SKD11 steel using WEDM, the main machining parameters influencing MRR are the discharge current and pulse-on time. In detail, MRR increases with the increase of discharge current and pulse-on time. It is noticeable that larger discharge current and longer pulse-on time mean higher pulse energy.

Figure 5 represents the relationship between the MRR and the energy input flux (Q_w_) which is defined as the amount of thermal energy input with unit area during unit time. It is shown that for each simulation group discerned by the pulse-on time (T_on_), MRR increases with the increase of Q_w_. For the two groups with T_on_ of 100 ps and 150 ps, respectively, the relationship is the same to a great extent, while for the one with T_on_ of 30 ps, compared to the other two, MRR is lower for the same Q_w_. In effect, larger Q_w_ results in the consequence that, in unit time, more energy is deposited and more atoms newly gain E_k_ higher than E_C_, thus MRR increases. Nevertheless, because of that, the quantities for the variables are all ones averaged over the T_on_, and thermal energy is not temporally linearly input, in effect, much quickly at the starting 10 ps. Q_w_ decreased with increasing T_on_ at the same application temperatures in all three cases. In addition, several picoseconds are needed for atoms capable of escaping to be removed. However, at the very end of the discharge, the melted area becomes cooled, and a portion of atoms about to escape fails, which will lead to a lower MRR. Moreover, the shorter T_on_ is, the more severe the impact. These two reasons led to the results as shown in Figure 5.

The dependencies of the energy efficiency (*β*) and the energy area density (EAD) as shown in Figure 6. The relationship between energy density and energy efficiency varies with the pulse width. Under different pulse width conditions, the energy efficiency increases rapidly first and then decrease slowly with the increase of energy density. Besides, for the same simulation group, *β* increases with the applied temperature (corresponding to the EAD) at first and decreases when the applied temperature is bigger than 7243 K. For the former result, lowering effect on the *β* due to the need of thermal energy for the cold material to melt becomes weaker when the T_on_ increases, and as the T_on_ increases large enough, the lowering effect is very subtle. While for the latter result, it can be explained as follows. A portion of the input thermal energy (*α*) is used to remove the surface material and (*γ*) to heat the left material. According to the Maxwell velocity distribution rule, at low temperature, N_r_ is rather small, thus *α*≪*γ*. As the applied temperature increases, N_r_ rises sharply, and *α* increases. Nevertheless, when the applied temperature is large enough, even it keeps increasing, and the relative increasing ratio of N_r_ is small. On the contrary, the larger the surface temperature is, the larger the gap between the surface temperature and the temperature at the inner part, and the quicker the speed at which input thermal energy is transferred to the vast cold matrix, thus, the larger the *γ* is, and consequently, *β* drops.

In the above analysis, the dependence of performance of micro-EDM on the EAD and Q_w_ is studied. Considering that Q_w_ = EAD/T_on_ and T_on_ is usually taken as the factor to study EDM, therefore, EAD and T_on_ are used as the dependent variables to construct the GPR model which is used to describe the relationship between the performance of micro-EDM and machining parameters in the next section.

## 5. GPR-Based Process Model for Micro-EDM

A non-parametric Bayesian regression technique is utilized in the GPR [26,27,28]. The GPR model has several promising features: the measure of confidence, a small number of training parameters, and different possibilities of prior knowledge. The GPR model was first used in the WEDM process optimization by Yuan [29]. In Yuan’s study, he used an EDM data set [30], and carried out an optimization on the EDM process that investigated the MRR with the effect of current, on-time, and off-time. Then, it was found that the performance of the GPR modeling was better than that of BPNN. To quantitatively measure the prediction accuracy of the GPR, the following functions are defined as Equations (6) and (7).

1. The average relative error

(6) $$e=\frac{1}{n}\sum_{1}^{n}|y_{i}^{*}-y_{i}|\times \frac{100}{y_{i\;\;}}$$

2. The maximum relative error

(7) $$mre=\max\left(|y_{i}^{*}-y_{i}|\times \frac{100}{y_{i}}\right)$$

**Figure 7:**
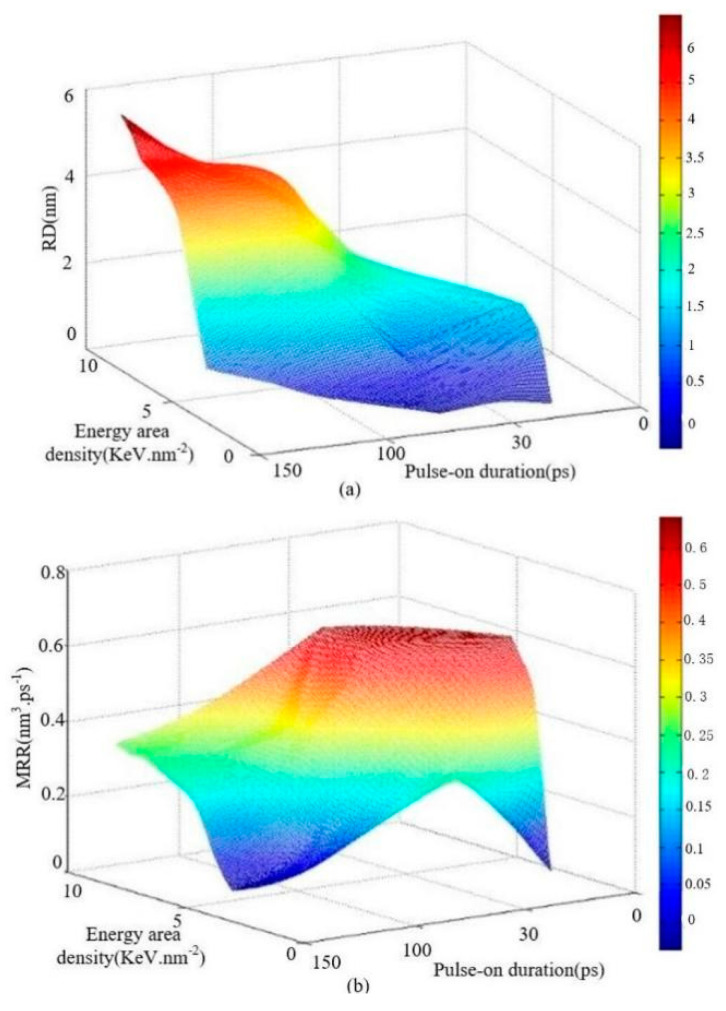
The relationships of machining parameters as input parameters, and RD or MRR as output response in the Micro-EDM model; (**a**) the effect of energy area density and pulse-on duration on RD; (**b**) the effect of energy area density and pulse-on duration on MRR.

It is note that y^*^_i_ is the *i*_th_ sample’s prediction value, *y*_i_ is the *i*_th_ sample’s measure value. In this study, the data set (Table 1) was split stochastically into 12 training samples and 3 test samples, and then it was modeled by GPR, in which the Matern class with type equal 3, the Matern covariance function with linear bias equal to 65/2 and number of degrees of freedom equal to 4, constant mean function with mean equal to 1 and Gaussian link function with noise standard deviation equal to 0.1 were used. Table 2 shows the relative error of the micro-EDM process, which is predicted by GPR. The average error (*e*) of RD and MRR are 3.33% and 5.26%, respectively; the maximum relative error (*mre*) of RD and MRR are 6.32% and 6.78%, respectively. Therefore, the micro-EDM process modeled by GPR is valid, since both the *e* and *mre* are less than 10%. Then, for the selection of optimum parameters, the GPR model can be used for a micro-EDM machining process to save the computing time of MDS. Figure 7 shows the two interaction effects of both EAD and T_on_ on RD and MRR. Through observing and analyzing the surface plots (Figure 7a), it is apparent that EAD and T_on_ have a remarkable interaction effect on the response variable, RD, when EAD is high and T_on_ is long, for the reason that the surface plot curves seriously and the change trend of the surface plot is extremely steep. On the contrary, the interaction effect is less obvious between them when EAD is low and T_on_ is short. Then, when EAD is high and T_on_ is long, the interaction between them is strong.

T_on_ has a remarkable interaction effect on the response variable MRR when EAD is high and T_on_ is short. On the contrary, the interaction effect is less important between them when EAD is high and T_on_ is long. The explanation of this phenomenon may be that response variable MRR is not only related to the RD, but is also related to the T_on_.

## 6. Experimental Verification

The validity of the research results presented in this paper is discussed by comparing them with those in academia on the precision EDM mechanism. This section mainly discusses the view of energy input intensity and crater shape.

### 6.1. Energy input Intensity

The energy input intensity directly affects the dynamic behavior and microstructure evolution of materials and then determines the removal process of materials. In this paper, the control variables (hot bath temperature T_HB_, current density *ρ*_e_, and energy input flux Q_w_) are selected based on the actual processing conditions, and the energy input intensity obtained is consistent with the actual situation.

**Figure 8:**
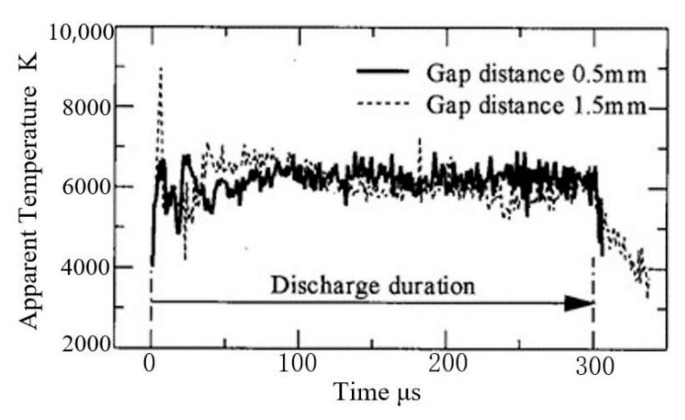
Evolution of the temperature in the discharge channel with time during in-gas discharge [31].

For the cathode material, the model energy input control variables are T_HB_ and *ρ*_e_, where T_HB_ is between 3622 K and 20,280 K, and *ρ*_e_ is from 0.2 × 10^12^ A.m^−2^ to 8 × 10^12^ A.m^−2^. The two input variables are assumed to be constant during the discharge process. Wataru et al. [31] measured the temperature distribution in the discharge channel during in-gas discharge machining (as shown in Figure 8), and it can be seen that the temperature in the discharge channel is relatively stable in the discharge process, with an average value of about 6000 K. Under other conditions, the discharge channel temperature can reach 20,000 °C [32]. The *ρ*_e_ ranges from 10^8^ A.m^−2^ to 10^9^ A.m^−2^ in macro-EDM [1]. However, in precision EDM, the discharge channel diameter ranges from microns to sub-microns, and the *ρ*_e_ increases greatly. For example, in the study of Kumar et al. [33] on nano-EDM technology, when W is used as the cathode, *ρ*_e_ reaches 1.3 × 10^13^ A.m^−2^. It can be seen that the range of control parameters selected in this paper is within the range of actual machining conditions.

For anode materials, the control variable is the energy input flux Q_w_. The Q_w_ in the simulation ranges from 10^14^ W.m^−2^ to 10^15^ W.m^−2^, while in the nano-EDM experiment conducted by Kumar et al. [33], the Q_w_ is 2.60 × 10^15^ W.m^−2^. Therefore, from the perspective of energy input intensity, the simulation experiment in this paper is comparable to the actual machining process. In the simulation model, water is used as the discharge liquid, and the central temperature of the discharge channel obtained is between 25,000 K and 40,000 K. According to theoretical calculation, the temperature in the discharge channel can reach 60,000 K when water is used as the discharge liquid [34], which is higher than the central temperature of the discharge channel in this paper. Considering the influence of discharge time and electrode material, the results in this paper are roughly consistent with current practice.

### 6.2. Crater Shape

**Figure 9:**
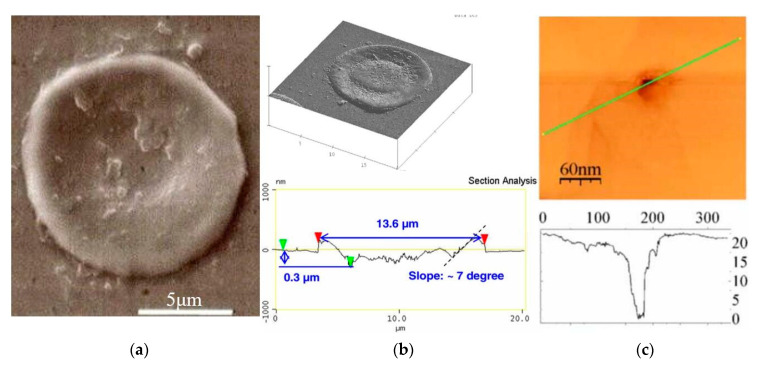
After electrical discharge machining of various materials, the morphology of voids: (**a**) Single crater shape of W material after micro-EDM processing [35]; (**b**) Single crater shape of H13 material after quasi-dry EDM processing [36]; (**c**) Single crater shape of Au material after nano-EDM processing [37].

**Figure 10:**
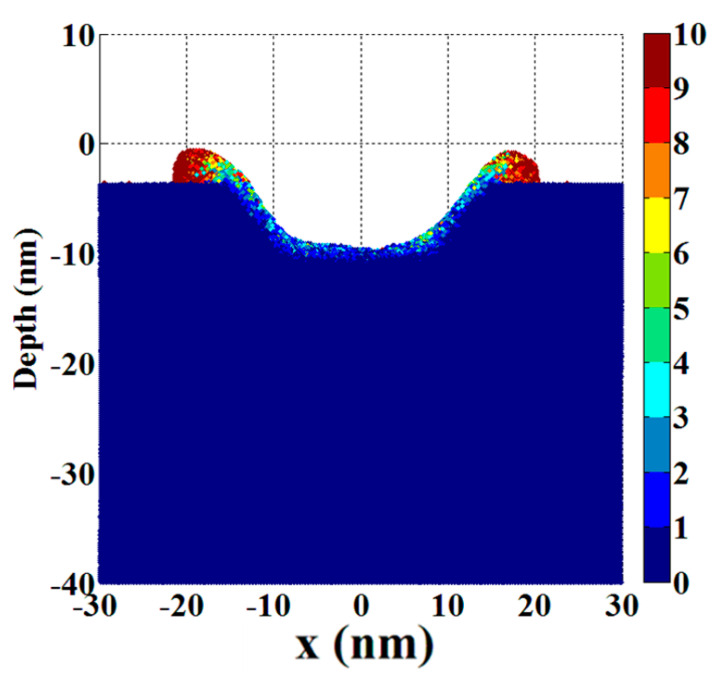
The crater shape formed by Cu cathode material in nano-EDM simulation under 150 ps and 9 KeV.nm^−2^.

The crater shape is the final result of material erosion caused by EDM, and the consistency between the simulated shape and the actual crater shape can also be used as one of the criteria to measure the accuracy of the simulation results. Figure 9 shows the crater shape in micro-EDM and nano-EDM machining, while Figure 10 shows the crater shape formed by Cu cathode material in nano-EDM simulation in this paper under 150 ps and 9 KeV.nm^−2^. It can be seen that in the actual processing, the bottom of the crater is rough and has many bumps. There are annular bumps outside the edge of the crater, and the view between the outer edge of the bump and the original electrode surface is right view or even acute view. In the simulation results presented in this paper, the bottom of the crater formed is uneven, and has a sharp peak due to incomplete erosion of the broken material and the inhomogeneity of the material removal process along the radial direction of the discharge channel. In addition, due to the gradual accumulation of erosion materials at the edge of the crater, the bulge formed by the accumulation keeps moving away from the axis of the discharge channel, so the outer edge of the bulge formed presents a right view or acute view with the surface of the original electrode. It can be seen that the crater shape simulated is close to the crater shape in actual precision EDM.

## 7. Conclusions and Outlooks

The following conclusions can be drawn from the present study.

(1) One hybrid intelligent process model, based on TTM-MDS and GPR, was proposed for micro-EDM to investigate the effect of machining parameters.
(2) There is a threshold of EAD to remove the atoms from the matrix surface. If considering the relationship between E_e_ and RD, there are optimal settings about RD and MRR.
(3) Using the GPR model of the micro-EDM machining process, the *e* of RD and MRR are 3.33% and 5.26%, respectively; the *mre* of RD and MRR are 6.32% and 6.78%, respectively.
(4) When EAD is high and T_on_ is long, there is an obvious interaction effect on RD between them. However, when EAD is high and T_on_ is short, there is an obvious interaction effect on MRR between them.

The temperature model established by molecular dynamics can not only study the removal mechanism of traditional metal materials in EDM, but can also be applied to ceramic materials [38]. The thermal physical model based on molecular dynamics to simulate temperature field, residual stress field, and deformation curve is considered to be an effective method to predict the performance of EDM, therefore, the effect of pulse discharge energy (pulse-on duration and current) on the deformation of EDM is studied [39,40,41]. A series of mechanisms of bubble generation and growth, and bubble movement and explosion are the core problems of precision manufacturing [42]. The successful application of dual-temperature methods combined with molecular dynamics (TTM-MD) in laser-bulk material interactions shows great potential in providing a panoramic picture of laser-nanoparticle interactions [43]. With the development of molecular dynamics, some common knowledge about EDM which has been widely accepted for a long time is also improving.

## Figures and Tables

**Table 1:** Experimental result on numerical modeling of the Micro-EDM process.

No.	Pulse Energy Density (KeV.nm^−2^)	Pulse-on Duration (ps)	Removed Depth (nm)	Removed Volume (nm^3^)	MRR (nm^3^.ps^−1^)
1	1.04	30	0.12	4.49	0.15
2	1.57	30	0.72	14.83	0.49
3	2.00	30	0.94	17.38	0.58
4	2.32	30	0.95	17.78	0.59
5	2.76	30	1.37	19.86	0.66
6	2.51	100	0.81	8.89	0.09
7	3.78	100	2.83	29.85	0.30
8	4.79	100	3.45	36.40	0.36
9	5.92	100	4.04	43.37	0.43
10	6.98	100	4.21	45.18	0.45
11	3.37	150	1.27	12.61	0.08
12	5.25	150	5.17	43.63	0.29
13	6.43	150	4.61	49.00	0.33
14	7.57	150	5.21	54.64	0.36
15	9.04	150	5.85	58.62	0.39

**Table 2:** The relative error of the Micro-EDM process predicted by GPR.

No.	Samples Measure Value		Predicted Value		Relative Error		Average Error	
	RD (nm)	MRR (nm^3^.ps^−1^)	RD (nm)	MRR (nm^3^.ps^−1^)	RD (%)	MRR (%)	RD (%)	MRR (%)
4	0.95	0.59	0.89	0.63	6.32	6.78		
8	3.45	0.36	3.35	0.38	2.90	5.56	3.33	5.26
12	5.17	0.29	5.21	0.28	0.77	3.45		
